# Carbon Nanotubes Reinforced Maleic Anhydride-Modified Xylan-g-Poly(N-isopropylacrylamide) Hydrogel with Multifunctional Properties

**DOI:** 10.3390/ma11030354

**Published:** 2018-02-28

**Authors:** Xinxin Liu, Tao Song, Minmin Chang, Ling Meng, Xiaohui Wang, Runcang Sun, Junli Ren

**Affiliations:** 1 State Key Laboratory of Pulp and Paper Engineering, School of Light Industry and Engineering, South China University of Technology, Guangzhou 510640, China; lxx19910312@163.com (X.L.); songt@scut.edu.cn (T.S.); 18322532189@163.com (M.C.); mengling0117@163.com (L.M.); fewangxh@scut.edu.cn (X.W.); 2 Beijing Key Laboratory of Lignocellulosic Chemistry, Beijing Forestry University, Beijing 100083, China; rcsun3@bjfu.edu.cn; 3 Key Laboratory of Pulp and Paper Science & Technology of Ministry of Education, Qilu University of Technology, Jinan 250353, China

**Keywords:** carbon nanotubes, xylan-based hydrogels, mechanical property, thermo-sensitive properties

## Abstract

Introducing multifunctional groups and inorganic material imparts xylan-based hydrogels with excellent properties, such as responsiveness to pH, temperature, light, and external magnetic field. In this work, a composite hydrogel was synthesized by introducing acid treated carbon nanotubes (AT-CNTs) into the maleic anhydride modified xylan grafted with poly(N-isopropylacrylamide) (MAX-g-PNIPAM) hydrogels network. It was found that the addition of AT-CNTs affected the MAX-g-PNIPAM hydrogel structure, the swelling ratio and mechanical properties, and imparted the hydrogel with new properties of electrical conductivity and near infrared region (NIR) photothermal conversion. AT-CNTs could reinforce the mechanical properties of MAX-g-PNIPAM hydrogels, being up to 83 kPa for the compressive strength when the amount was 11 wt %, which was eight times than that of PNIPAM hydrogel and four times than that of MAX-g-PNIPAM hydrogel. The electroconductibility was enhanced by the increase of AT-CNTs amounts. Meanwhile, the composite hydrogel also exhibited multiple shape memory and NIR photothermal conversion properties, and water temperature was increased from 26 °C to 56 °C within 8 min under the NIR irradiation. Thus, the AT-CNTs reinforced MAX-g-PNIPAM hydrogel possessed promising multifunctional properties, which offered many potential applications in the fields of biosensors, thermal-arrest technology, and drug-controlled release.

## 1. Introduction

Hydrogel is a gel-like material that consists of water-soluble polymers cross-linked physically or chemically. It swells by absorbing a great quantity of water rather than being dissolved in water [1,2]. Recently, functionalized hydrogels have attracted great interests in fabricating intelligent hydrogels (pH, temperature, magnetic, and light response), high strength hydrogels, self-healing hydrogels, and shape memory hydrogels [3,4,5,6,7]. Those intelligent hydrogels could show the potential application in drug release, adsorption, sensors, tissue engineering and so on.

In recent years, natural-occurring polymers have been intensively studied for fabricating intelligent hydrogels in drug release, adsorption and many other fields [8] due to their abundance in resource, low cost, biocompatibility, biodegradability, and non-toxicity. For example, chitosan hydrogel grafted with thermo-responsive poly(N-isopropylacrylamide) (PNIPAM) endowed it with temperature-triggered volume shrinkage and reversible swelling/de-swelling behaviors [9]. Starch-based hydrogels were prepared via the simultaneous formation of magnetic iron oxide nanoparticles and in situ radical solution polymerization of poly(acrylicacid-co-acrylamide) (P(AA-co-AM)) grafted onto starch backbones in the presence of graphene oxide (GO) nano sheets, which exhibited pH-responsiveness and magnetic characteristic on swelling/de-swelling pulsatile behaviors [10]. GO/polyacrylamide/aluminum ion cross-linked carboxymethyl hemicellulose nanocomposite hydrogels (GO/PAM/Al^3+^-CMH) were produced by the two-step process, which possessed the high strength and great elasticity [11]. The PAM/gelatin/polyvinyl alcohol (PVA) hydrogel with triple-network was prepared by a simple one-pot method, consisting of copolymerization, cooling and freezing/thawing [12], which displayed superior mechanical properties.

Xylan is the second-dominating polysaccharide after the cellulose and is linked by *β*-d-1,4-xyloside bond with a variety of side groups, which as the major hemicellulose is in grasses and in dicot secondary cell walls [13]. There are large amounts of free hydroxyl groups on the surface of xylan molecules that could provide the opportunity to be modified chemically by etherification, esterification, oxidation and grafting copolymerization to improve its reactivity [14,15,16]. For instance, maleic anhydride was used to react with hydroxyl groups of xylan to form maleic anhydride modified xylan (MAX), which contained both unsaturated double bonds and carboxyl groups with electronegative, hydrophilic and adsorption properties [17]. MAX could be applied as the raw material for fabricating new functional hydrogels, such as MAX-g-P(NIPAm-co-AA) hydrogel with pH response improved by the carboxyl groups and unsaturated double bonds on the MAX chains and controllable crosslinking density with different degree of substitution (DS) of MAX [18]. The MAX-PVA hydrogel was developed and could be designed to meet the swelling and strength requirements by varying MA and PVA contents in the hydrogel [19]. Therefore, MAX as a xylan derivative has an influence on properties of hydrogels such as the swelling behavior, response and strength.

Currently, nanotechnology and nanocomposites could create opportunities for fabricating nanomaterials. Carbon nanotubes (CNTs) are a kind of one-dimensional quantum material with nanoscale at radial dimension and micron grade at the axial dimension. CNTs have unique electrical characteristics that are beneficial for endowing composite materials with electrical conductivity [20,21,22]. For example, highly conductive polypyrrole (PPy)/CNT composites were synthesized by in-situ polymerization, and the maximum conductivity was 52 S/cm at a loading of 5 wt % of CNTs [23]. The addition of copper/CNTs hybrid also enhanced the conductivity of high-density polyethylene (HDPE)-matrix composite by 90% [24]. Besides, CNTs also have excellent mechanical properties because of strong covalent bonds existing between the carbon atoms that constitute the structure of CNTs. Therefore, CNTs have the superior ability as the additive to reinforce the mechanical properties of composites. Poly(acrylamide)-multiwalled carbon nanotubes (PAM-MWNTs) hydrogels were prepared through the radiation-induced polymerization and crosslinking of AM and MWNTs [25]. The compressive strength of PAM-MWNTs hydrogels was increased by 58%, compared to PAM gels. CNTs/epoxy, CNTs/polypropylene (PP) and CNTs/polyvinyl chloride (PVC) composites were produced with increased tensile strength by 28%, 18% and 7% compared to hydrogels made from neat epoxy, PP and PVC, respectively [26]. However, CNTs are stable and insoluble in many solvents, which inhibit its applications. Therefore, hydroxyl and carboxyl groups are usually introduced to CNTs by acid treatment to improve their reactivity [27].

The objective of this paper was to fabricating new intelligent MAX-based hydrogels possessing multifunctional properties such as high mechanical strength, electrical conductive, thermo-sensitive properties as well as the shape memory. These hydrogels were prepared by the cross-linking polymerization of MAX with NIPAm using *N*,*N*-methylene-bis-acrylamide (MBA) as the cross-linker and acid treated CNTs (AT-CNTs) as the reinforcment. The influence of AT-CNTs was discussed on the hydrogel structure, swelling ratio, mechanical strength, conductivity, and photothermal conversion properties. 

## 2. Materials and Methods

### 2.1. Materials

MAX was prepared according to our previous work (DS = 0.64) [17]. CNTs were purchased from Nanjing XFNANO Materials Technology (Nanjing, China). NIPAM (98%, with stabilizer hydroquinone monomethyl ether), ammonium persulfate (APS, 99%) and tetramethylethylenediamine (TMEDA, 99%) were obtained from Macklin Reagent Company Limited (Shanghai, China). MBA (98%) was supplied from Aladdin Reagent Company Limited (Shanghai, China). Ethanol was purchased from Guangzhou Chemical Reagent Factory (Guangzhou, China). All of the reagents and chemicals were AR grade and used as received. Deionized water was used in all experiments.

### 2.2. Acid Treatment of Carbon Nanotubes

2 g of CNTs were dispersed in 80 mL mixed acids in which the volume ratio of concentrated sulfuric acid to concentrated nitric acid was 3:1 and the mixture was treated by sonication for 8 h. After the treatment, the mixture was precipitated for 24 h followed by four times dilution with deionized water and dialyzed in water for seven days until pH reached to neutral. AT-CNTs were finally obtained after lyophilization [21].

### 2.3. Preparation of Composite Hydrogels

**Figure 1:**
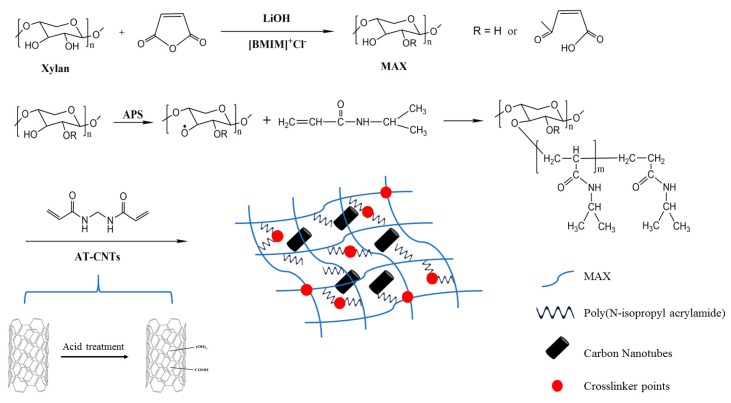
Synthesis process of MAX-g-PNIPAM/AT-CNTs composite hydrogel.

In this work, NIPAM (1.3–2.0 g) and a specific amount of MAX (0–0.7 g) were added in deionized water and stirred in the ice bath. Then different contents of AT-CNTs (the weight ratio to the total amount of NIPAM and MAX) were added into the mixture solution. The solution was sonicated and stirred. After the homogenous mixture was obtained, 0.05 g APS, 0.05 g MBA were added. After adding 6 μL of TMEDA to the mixture, the obtained homogenous solution was poured into molds to proceed further polymerization at 5 °C and finally the hydrogel was formed. The hydrogel was then soaked in deionized water to remove the impurities. The preparation conditions of the MAX-g-PNIPAM/AT-CNTs hydrogel are shown in Table 1. The total volume of solution was 20 mL, while the total amount of NIPAM and MAX was kept for 2 g. The synthesis process of MAX-g-PNIPAM/AT-CNTs composite hydrogels was proposed in Figure 1. 

### 2.4. Characterization

The changes of bonds in CNTs and AT-CNTs were characterized using Raman Spectrometer (RENISHAW 2000, London, UK) equipped with a Spectra-Physics Ar: Kr laser source for an excitation at 532 nm.

The Fourier transform infrared spectroscopy (FT-IR) spectroscopies of CNTs, AT-CNTs, MAX, xylan and hydrogels were determined by Vertex 33 spectrophotometer (Bruker, Karlsruhe, Germany). All samples were dehydrated in an oven at 50 °C before testing, and then the finely ground samples were mixed with KBr pressed into a plate for the measurement.

The morphology of AT-CNTs was observed using a transmission electron microscopy (TEM) (FEI Tecnai G2 F20 S-TWIN, Hillsboro, OR, USA). AT-CNTs were dispersed and diluted in ethanol, followed by ultrasonic treatment before drying on a copper wire.

The X-ray diffraction (XRD) patterns of CNTs, AT-CNTs, MAX, and hydrogels were collected by D8 ADVANCE X-ray diffractometer (Bruker, Karlsruhe, Germany) at a speed of 2° per minute and a range of 2θ = 10–50°. The hydrogel was lyophilized and ground into a powder prior to the test.

The morphology of hydrogels was observed by scanning electron microscopy (SEM) (Hitachi S3700, Tokyo, Japan) with an accelerating voltage of 10 kV. The hydrogels were dehydrated by vacuum freeze-drying before testing.

A simultaneous thermal analyzer (TGA Q500, TA Instruments, New Castle, DE, USA) under a nitrogen flow of 20 mL/min was applied to determine the thermogravimetric property of the hydrogels. 10 mg dried hydrogel sample was ground to powder and heated from 25 °C to 700 °C at a 10 °C/min heating rate in an open alumina crucible.

The compressive strength of the hydrogel was measured by Tensile Compression Material Testing Machine (INSTRON 5565, Boston, MA, USA). The circular hydrogel was swelled at room temperature to achieve swelling equilibrium before testing.

Swelling ratio was tested by the immersion of the dried hydrogels in deionized water until their weight became constant. The hydrogels were then removed from the water and weighed on balance after removing the excess water by gently tapping the hydrogel sample with filter paper. The swelling ratio was calculated using the following equation:

(1) $$\mathrm{Swelling}\;\mathrm{Ratio}\;\left(\mathrm{SR}\right)=\frac{W_{s}-W_{d}}{W_{s}}$$

where W_d_ and W_s_ represent the weights of the dried hydrogel and the hydrogel at swelling equilibrium state, respectively.

The low critical solution transition (LCST) of the swollen hydrogels was measured by a differential scanning calorimeter (DSC) with a heating rate of 5 °C/min under a nitrogen atmosphere (25.0 mL/min nitrogen flow rate) and a thermal analysis temperature range from 15 °C to 55 °C. The hydrogel was swelled at room temperature to achieve swelling equilibrium before testing.

The electrical conductivity of the hydrogels was measured at room temperature while using the FT-340 four-point probe method (Ningbo, China). The hydrogel was swelled at room temperature to achieve swelling equilibrium before testing.

### 2.5. Photothermal Properties of Hydrogels

The hydrogel was placed in a cuvette containing deionized water which were divided into experimental group (E) and control group (C). The near-infrared (NIR) laser source was then directed at the experimental group and irradiated at a distance of 5 cm. The NIR laser source has a power of 2 W and a wavelength of 808 nm. After radiation of different times by a NIR laser, the water temperature in the cuvette was immediately measured with a temperature probe.

### 2.6. Shape Memory Effect of Hydrogels

The rectangular MAX-g-PNIPAM/AT-CNTs hydrogel (gel-13) was placed in a 10% NaCl solution at room temperature to obtain a definite shape, and then placed in deionized water to observe the shape changes.

## 3. Results and Discussion

### 3.1. Characterizations of AT-CNTs

**Figure 2:**
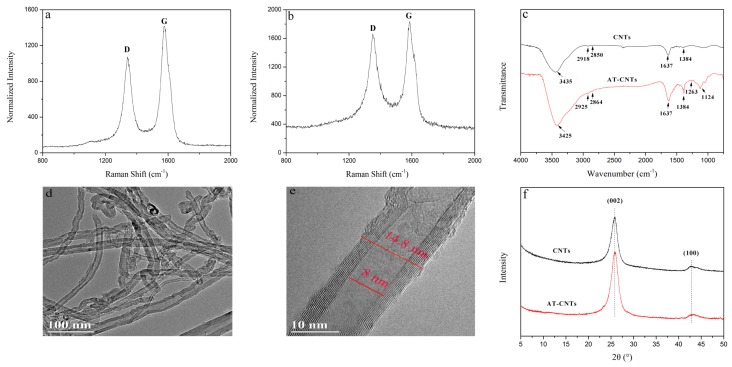
Characterizations of CNTs and acid treated carbon nanotubes (AT-CNTs), Raman spectra of CNTs (**a**) and AT-CNTs (**b**); FTIR spectra of CNTs and AT-CNTs (**c**); transmission electron microscopy (TEM) images of AT-CNTs (**d**,**e**) and X-ray diffraction (XRD) of CNTs (**a**) and AT-CNTs (**f**).

The changes of the bonds in CNTs and AT-CNTs were determined by Raman spectra and illustrated in Figure 2a,b, respectively. In Figure 2a,b, the D band reflects the defects as well as the disorder, while G band is generated by the stretching motion of all the sp^2^ atoms in the carbocyclic or long chain. The relative intensity (I_D_/I_G_) reflects the degree of disorder and defect density of the sample, and the large the ratio could lead to the high the sample disorder and defect density [28]. The corresponding peak center and intensity of the CNTs and AT-CNTs were shown in Table 2. The I_D_/I_G_ ratio of AT-CNTs was higher than that of CNTs, indicating that the ordered graphite structure in the CNTs was destroyed and replaced by the functionalized AT-CNTs [29].

FTIR spectra in Figure 2c show that both CNTs and AT-CNTs have absorption bands located at 3425, 2925, 2864, 1637, and 1384 cm^−1^. The absorption band at 3425 cm^−1^ is owing to the stretching vibration of -OH groups, while the adsorption bands at 2925 and 2864 cm^−1^ represent the stretching vibrations of aromatic and aliphatic C-H, respectively [30]. After acid treatment, the intensity of -OH group was enhanced. The absorption band of C=O stretching vibration is located at 1637 cm^−1^ and the symmetric shear vibration of C-H is at 1384 cm^−1^. However, besides those bands, there are also two bands at 1263 and 1124 cm^−1^ on the AT-CNTs spectra that are related to the stretching vibration of phenolic C-O and alcoholic C-O, respectively. This implied that carboxyl and hydroxyl groups were successfully introduced to the surface of CNTs after acid treatment [31].

The TEM micrographs of AT-CNTs were exhibited in Figure 2d,e. A tubular structure of AT-CNTs with diameter about 14.8 nm was observed. There is the observation of little deformation on the surface of CNTs after acid treatment, which was different from the smooth surface of pure CNTs [32,33].

XRD patterns of CNTs and AT-CNTs were shown in Figure 2f. Obviously, the crystalline structure of CNTs did not change after acid treatment. They had the same diffraction peaks at 25° and 42°, corresponding to (002) and (100) orientations, respectively [34], implying that its *d*-spacing resembles the one of pristine graphite [35].

### 3.2. Characterizations of Hydrogels

**Figure 3:**
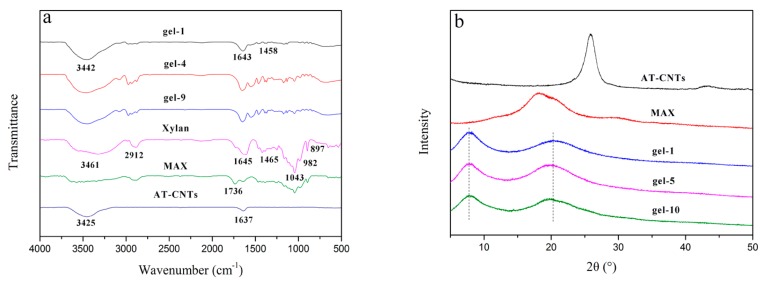
FTIR spectra of xylan, MAX, AT-CNTs and hydrogels (**a**) and XRD pattern of MAX, AT-CNTs and hydrogels (**b**).

FTIR spectra of MAX, AT-CNTs, and MAX-g-PNIPAM/AT-CNTs dried hydrogels were illustrated in Figure 3a. The characteristic absorption bands of xylan as well as MAX in the spectrum are 3461, 2912, 1645, 1465, 1043, 982, and 897 cm^−1^ [36]. A broad adsorption bands at 3461 cm^−1^ is for the stretching of -OH groups, and the C-H stretching vibration band occurs at 2912 cm^−1^. The adsorption bands at 1465 and 1043 cm^−1^ are the stretching vibration bands of -C-H and -C-O-C-, respectively. Arabinose units and *β*-glucoside linkage are represented by the adsorption bands at 982 cm^−1^ and 897 cm^−1^. The absorption band at 1736 cm^−1^ in the spectrum of MAX is the characteristic absorption band of ester group (C=O), which is indicative of the successful modification of xylan by maleic anhydride [17].

In FTIR spectrum of PNIPAM (gel-1), the stretching vibration band at 1643 cm^−1^ is derived from the C=O of NIPAM, and the asymmetric adsorption band of COO^−^ appears at 1458 cm^−1^ [37]. In the spectrum of MAX-g-PNIPAM (gel-4), the -OH stretching of MAX and PNIPAM occurs at 3460 cm^−1^. In addition, C-H stretching vibration band of MAX is observed at 2933 cm^−1^, and the asymmetric adsorption band of COO^−^ of NIPAM appears at 1461 cm^−1^. In the spectrum of MAX-g-PNIPAM/AT-CNTs (gel-9), the characteristic bands of MAX and NIPAM are observed and the phenolic C-O stretching vibration and the alcoholic C-O stretching vibration of AT-CNTs are present at 1277 cm^−1^ and 1131 cm^−1^, respectively [31]. All of these characteristic bands appeared clearly in FTIR spectra, indicating the successful synthesis of hydrogels.

Figure 3b shows the XRD patterns of AT-CNTs, MAX, and MAX-g-PNIPAM/AT-CNTs dried hydrogels. The gel-1, gel-5, and gel-10 exhibited two broad diffraction peaks at 2θ = 7.9° and 2θ = 19.97° which implied the amorphous structure of PNIPAM [38]. In patterns of hydrogels, there were no sharp peaks of AT-CNTs observed in formed hydrogels due to the hydrogen bonding among the functional groups of AT-CNTs and other functional groups of MAX and PNIPAM during the formation of the hydrogel [39]. This also showed that AT-CNTs changed from crystalline to amorphous due to the formation [40].

### 3.3. Equilibrium Swelling Ratio of Hydrogels

**Figure 4:**
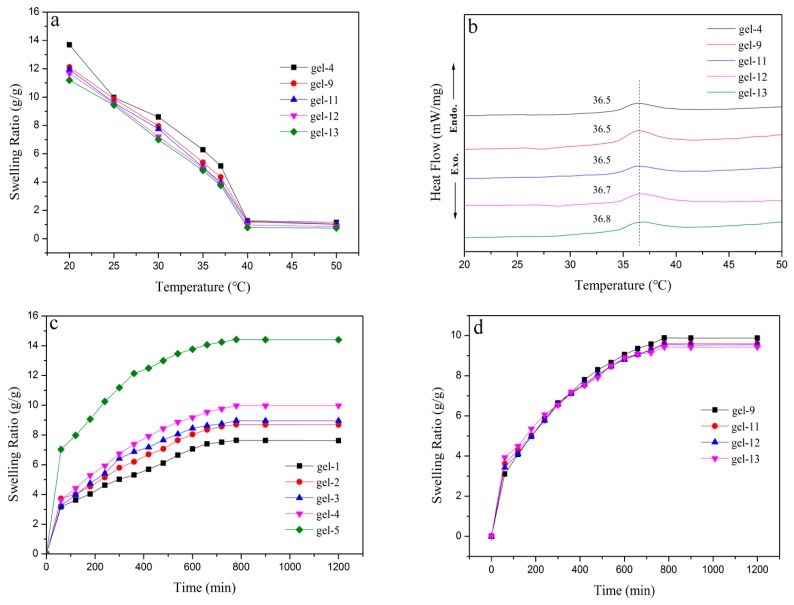
The swelling behavior (**a**,**c**,**d**) and differential scanning calorimeter (DSC) (**b**) curves of hydrogels.

The swelling behaviors and DSC curves of hydrogels were illustrated in Figure 4. The equilibrium swelling ratio of these hydrogels decreased with the increase of temperatures (Figure 4a). A sharp decrease of the swelling ratio was found between 35 °C and 40 °C, indicating that these hydrogels started to the volume shrink when the temperature was higher than 35 °C. This could be explained that when the temperature was higher than the low critical solution temperature (LCST) of NIPAM, the intermolecular hydrogen bonds in hydrogel were destroyed and intramolecular hydrogen bonds started to play the dominating role and resulted in the shrinkage [41].

The Figure 4b shows the DSC curves of hydrogels samples with (gel-9, gel-11, gel-12 and gel-13) or without AT-CNTs (gel-4). Obviously, hydrogels with or without AT-CNTs had the higher LCST than that pure PNIPAM hydrogels (about 32 °C [42]), indicating that the addition of MAX and AT-CNTs had an important effect to the temperature response of PNIPAM hydrogels. The LCST was increased with the enhancement of AT-CNTs amounts. The LCST was up to 36.8 °C when the amount of AT-CNTs was 11% (gel-13). This was because the hydrogels with the addition of AT-CNTs generated more hydrogen bonds with H_2_O, which needed more energy to be destroyed [43]. 

The equilibrium swelling ratio of the hydrogels as the functions of MAX amounts in hydrogels was shown in Figure 4c. The equilibrium swelling ratios of hydrogels were increased with the increase of the MAX amounts, and the equilibrium swelling ratio of hydrogel (gel-5) reached up to 14.41 g/g when the amount of MAX was 0.7 g (Table 1). This was probably due to the increase of hydrogel hydrophilicity that resulted from the addition of MAX, making the increase of equilibrium swelling ratio.

The effects of AT-CNTs addition on the equilibrium swelling ratio was exhibited in Figure 4d. There was no significant change as the increase of AT-CNTs amounts that was similar to the swelling ratio of hydrogels without AT-CNTs (gel-4 in Figure 4c). With the AT-CNTs amount increased from 2% to 11%, the equilibrium swelling ratio of four samples were 9.87, 9.59, 9.54 and 9.43 g/g, respectively. The only slight decrease was observed. The hydroxyl and carboxyl groups were produced on the surface of AT-CNTs after acid treatment that could enhance the interaction between AT-CNTs and MAX-g-PNIPAM chains and endowed the hydrogel with high crosslinking density, resulting in the reduction of the equilibrium swelling ratio hydrogels.

### 3.4. Thermal Stability of Hydrogels

**Figure 5:**
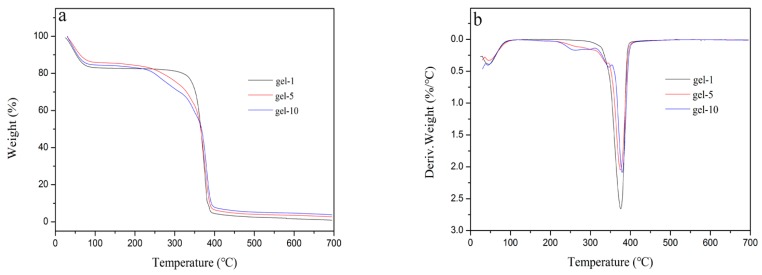
The TG curve trends (**a**) and DTG curve trends; (**b**) of hydrogels.

The thermal stability of PNIPAM hydrogel (gel-1), MAX-g-PNIPAM hydrogel (gel-5) and MAX-g-PNIPAM/AT-CNTs hydrogel (gel-10) were showed in Figure 5. The TG curve trends of three hydrogels were similar and all showed three degradation steps (Figure 5a). The weight loss happened before 220 °C, which was owing to the degradation of small molecules and water evaporation. The primary weight loss of hydrogels appeared in the range of 220-400 °C, this mainly resulted from the breaking of intermolecular hydrogen bonds and the molecular side chains of component in the gels [36]. After 400 °C, the carbonation of the polymer matrix started. The initial decomposition temperature of gel-5 and gel-10 were 224 °C and 232 °C, respectively, which was lower than 276 °C of gel-1. Above 363 °C, gel-5 and gel-10 had the lower thermal stability than gel-1. After that, the opposite trend was observed. It was due to the difference in the formed three-dimensional network structure of hydrogels.

The same phenomena were seen also from the results of DTG (Figure 5b). From the curve of DTG, the weight loss of hydrogels was kept at a constant rate before 220 °C which indicated that the water absorbed in the hydrogel was evaporated after 100 °C. The weight loss rate of these hydrogels increased sharply in the range of 220–400 °C. When the rate was increased to the maximum, the peaks then appeared in gel-1, gel-5, and gel 10 curves corresponding to the temperature at 375 °C, 376 °C, and 378 °C. When the temperature was higher than 400 °C, the rate of weight loss was constant [44].

### 3.5. Mechanical Properties of Hydrogels

**Figure 6:**
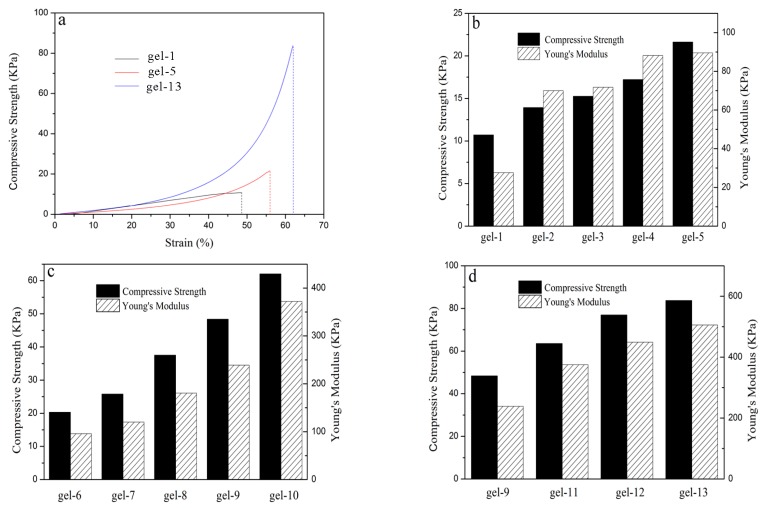
Compressive strength of the PNIPAM hydrogel with/without MAX and AT-CNTs (**a**); compressive strength and Young’s modulus of the MAX-g-PNIPAM hydrogels (**b**); compressive strength and Young’s modulus of the MAX-g-PNIPAM/AT-CNTs hydrogels (**c**,**d**).

The mechanical tests, e.g., compressive strength as well as Young’s modulus of hydrogels were shown in Figure 6. Figure 6a shows the compressive strength of the PNIPAM hydrogel with/without MAX and AT-CNTs. The PNIPAM hydrogel (gel-1) was broken at a compressive deformation of 48% with a maximum compressive strength of 10.72 kPa. However, MAX-g-PNIPAM hydrogel displayed the compressive strength twice as high as that of the PNIPAM hydrogel, being 21 kPa at a compressive deformation of 55%. Furthermore, when AT-CNTs was added to the MAX-g-PNIPAM hydrogel, the compressive strength reached to 83 kPa with a compressive deformation of 62%, which was eight times as high as that of the PNIPAM hydrogel. Thus, MAX and AT-CNTs could obviously enhance the compressive strength of PNIPAM hydrogel.

The compressive strength and Young’s modulus of the hydrogel increased with an increase of the MAX amount in MAX-g-PNIPAM hydrogel (Figure 6b). When the amount of MAX was increased from 0 to 0.7 g, the compressive strength of hydrogel was enhanced from 10.72 kPa to 21.64 kPa, while the Young’s modulus was improved from 27.6 kPa to 89.52 kPa. This indicated that MAX showed the positive impact on improving the mechanical properties of hydrogels, probably due to the formation of a stronger three-dimensional network structure resulted from the addition of MAX (Figure 1).

The addition of MAX and AT-CNTs further increased the mechanical properties of MAX-g-PNIPAM hydrogels in Figure 6c,d. When the amount of AT-CNTs was 2%, the mechanical properties of hydrogels were mainly affected by the amounts of MAX in MAX-g-PNIPAM/AT-CNTs hydrogels. Both compressive strength and Young’s modulus of the MAX-g-PNIPAM/AT-CNTs hydrogels were increased by 1.9–2.8 times than that of the corresponding MAX-g-PNIPAM hydrogels (Figure 6b), up to 60 kPa for compressive strength and 375 kPa for Young’s modulus when the amount of MAX was 0.7 g in MAX-g-PNIPAM/AT-CNTs hydrogel. This could be explained by the introduction of AT-CNTs which endowed hydrogels with higher cross-linked density, and consequently resulted in an increase for the mechanical properties. Another reason would be ascribed to the naturally existing strong mechanical property of CNTs that was inherited to the MAX-g-PNIPAM/AT-CNTs composite hydrogel.

It was found that AT-CNTs started to play an important role for the mechanical properties of hydrogels (Figure 6d). Both compressive strength and Young’s modulus increased gradually as the increase of the AT-CNTs amounts. The maximum mechanical properties were observed in this work and was 83.73 kPa for the compressive strength and 505 kPa for Young’s modulus when the amount of AT-CNTs was 11 wt %. AT-CNTs containing hydroxyl and carboxyl groups was beneficial to interact with the MAX-g-PNIPAM network through hydrogen bonds to form denser structures, consequently causing a remarkable reinforcement of the mechanical properties of PNIPAM hydrogel.

### 3.6. Morphology of Hydrogels

**Figure 7:**
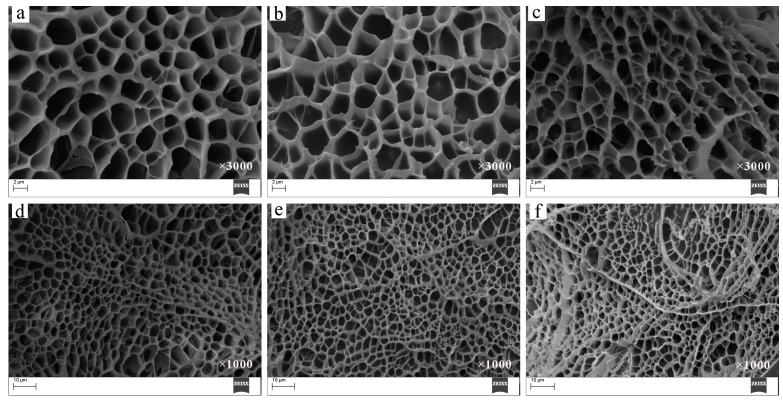
SEM images of gel-1 (**a**,**d**), gel-3 (**b**,**e**), and gel-8 (**c**,**f**).

Morphologies of the hydrogels were determined by SEM and a homogeneous network structure was observed for PNIPAM hydrogels (gel-1, Figure 7a,d), MAX-g-PNIPAM (gel-3, Figure 7b,e) and MAX-g-PNIPAM/AT-CNTs (gel-8, Figure 7c,f), which is presented below in Figure 7. The PNIPAM and MAX-g-PNIPAM hydrogels displayed similar network structure, though the compression strength and elasticity of the MAX-g-PNIPAM hydrogel was higher due to the occurring of MAX. This was because MAX played more important roles for the mechanical properties of MAX-g-PNIPAM hydrogels than PNIPAM did alone. The image of MAX-g-PNIPAM/AT-CNTs hydrogel illustrated smaller pore size and denser network structure than other two hydrogels because of the introduction of AT-CNTs, which was beneficial for improving the mechanical properties of the obtained hydrogel materials. This is in accordance with the previous results of mechanical properties.

### 3.7. Conductivity of Hydrogels

**Figure 8:**
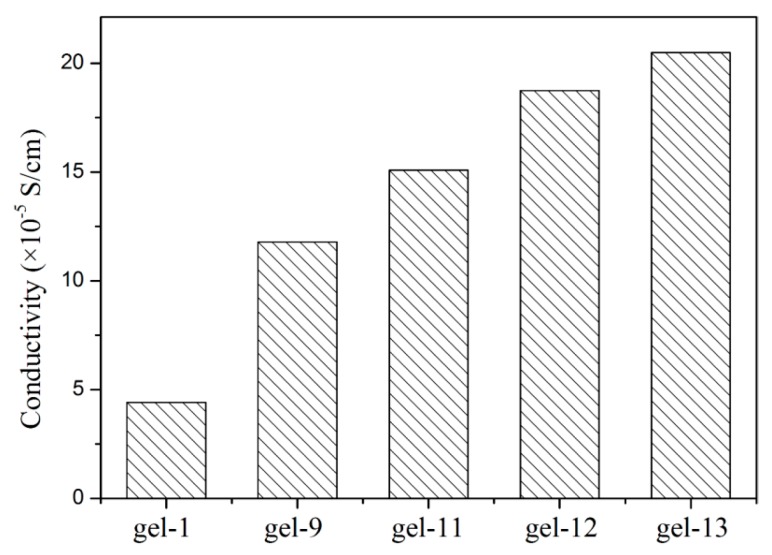
The conductivity of the hydrogels.

The electrical conductivity of the hydrogels was measured and displayed in Figure 8. Obviously, the electrical conductivity was improved with increasing of AT-CNTs amounts due to the function of AT-CNTs. When the amount of AT-CNTs was 11%, the corresponding electrical conductivity reached up to 20 × 10^−5^ S/cm. It was mentioned that the increasing of CNTs amounts resulted in the improvement of the electrical conductivity [45]. The difference in the electrical conductivity was attributed to the amounts and structure of CNTs and the structure of materials. The addition of AT-CNTs was not enough to form a complete conductive network channel in the hydrogel due to the large interval between AT-CNTs.

### 3.8. Photothermal Conversion of Hydrogel

**Figure 9:**
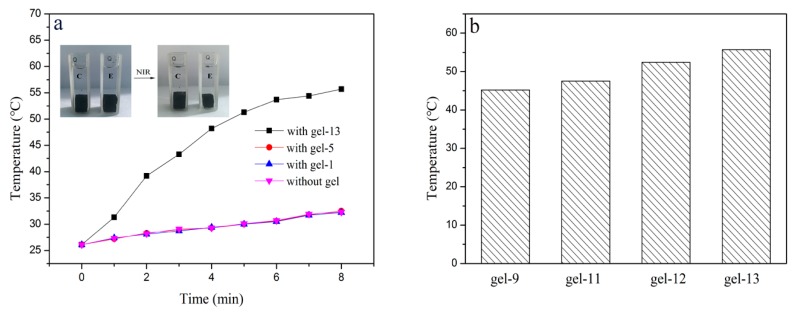
The photothermal conversion of hydrogels (**a**) and the temperature of hydrogels containing AT-CNTs after 8 min irradiation time (**b**).

The photothermal conversion of hydrogels was studied in Figure 9. There was no change observed for hydrogel in the control group (C) without NIR laser (Figure 9a), however, the phase transition of hydrogel in the experimental group (E) was found after 2 min irradiation (Figure 9a). The hydrogel returned to its original state after removing the NIR laser and placing at room temperature for a period of time.

As shown in Figure 9a, the temperature of the water with hydrogel was increased with the irradiation time. The irradiation time affected the temperature change of the hydrogels, but to different scales. The temperature of the water in cuvette of PNIPAM (gel-1), MAX-g-PNIPAM (gel-5) hydrogels was increased slightly from about 26 °C to 32 °C after 8 min irradiation time. The same observation was obtained when irradiating pure water without hydrogel in cuvette. On the contrary, the water temperature in the cuvette increased significantly from about 26 °C to 56 °C when AT-CNTs was involved in the hydrogel, and increased gradually with the increase of the AT-CNTs amounts after 8 min irradiation time (Figure 9b). No obvious change in temperature was observed with water, PNIPAM (gel-1) and MAX-g-PNIPAM(gel-5) hydrogels, indicating that the temperature of hydrogel increased more highly than its LCST, probably due to the photothermal effect originated from the function of black-colored AT-CNTs [46].

### 3.9. Shape Memory Characterization of Hydrogel

**Figure 10:**
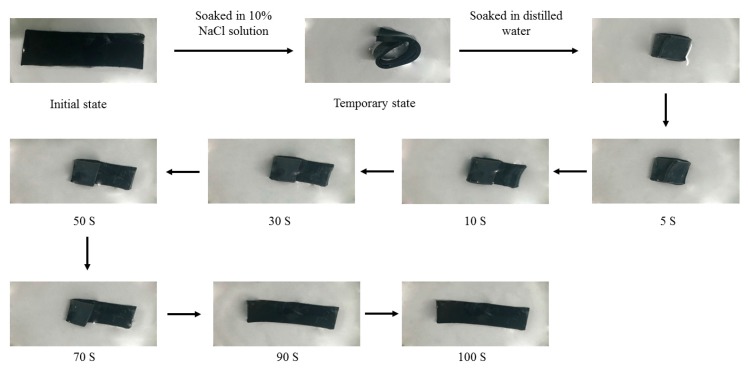
Shape memory behavior of MAX-g-PNIPAM/AT-CNTs hydrogel (gel-13).

Figure 10 displays the shape memory behavior of MAX-g-PNIPAM/AT-CNTs hydrogel (gel-13). Gel-13 was crimped in the 10% NaCl solution and recovered to the initial shape step by step 100 s after immersion in deionized water at room temperature. Park and Hoffman had found that NaCl induced an abrupt volume phase transition in the PNIPAM gel, while the LCST of the gel was lowered by increasing the concentration of NaCl solution [47]. Therefore, when gel-13 was soaked in the NaCl solution, the LCST of hydrogel was decreased which caused the phase transition of the hydrogel, followed by dehydration and shrinkage to form a definite curved shape. When the hydrogel was placed in deionized water, the hydrogel absorbed water quickly and recovered to its original shape after a short period of time. Thus, the salt-induced phase transition of MAX-g-PNIPAM/AT-CNTs hydrogel would have potential applications, such as biosensors.

## 4. Conclusions

In this work, AT-CNTs reinforced MAX-g-PNIPAM hydrogel was successfully synthesized with multifunctional properties, such as electrical conductivity, photothermal conversion ability, and shape memory. The mechanical properties of the hydrogel were sharply increased by the addition of AT-CNTs. When the amount of AT-CNTs addition was 11%, the compressive stress of hydrogel reached to 83 kPa, and the conductivity reached to 20 × 10^−5^ S/cm. Meanwhile, after irradiating by NIR, the temperature in cuvette with MAX-g-PNIPAM/AT-CNTs hydrogel increased to 55 °C after 8 min of irradiation, indicating that the hydrogel displayed the ability of photothermal conversion. Moreover, hydrogels exhibited multiple shape memory property. The obtained CNTs-reinforced MAX-g-PNIPAM hydrogel would have potential applications in the fields of biosensors, thermal-arrest technology and drug controlled release.

## Figures and Tables

**Table 1:** Preparation condition of the maleic anhydride modified xylan grafted with poly(N-isopropylacrylamide) (MAX-g-PNIPAM/AT)-carbon nanotubes (CNTs) hydrogels.

Sample	NIPAM (g)	MAX (g)	AT-CNTs (wt %)
gel-1	2	0	0
gel-2	1.9	0.1	0
gel-3	1.7	0.3	0
gel-4	1.5	0.5	0
gel-5	1.3	0.7	0
gel-6	2	0	2
gel-7	1.9	0.1	2
gel-8	1.7	0.3	2
gel-9	1.5	0.5	2
gel-10	1.3	0.7	2
gel-11	1.5	0.5	5
gel-12	1.5	0.5	8
gel-13	1.5	0.5	11

**Table 2:** Peak center (υ) and intensity (I) of the CNTs and AT-CNTs

Sample	υ_D_ (cm^−1^)	υ_G_ (cm^−1^)	I_D_	I_G_	I_D_/I_G_
CNTs	1340.51	1579.16	1075.84	1412.12	0.76
AT-CNTs	1350.8	1585	1665.2	1836.3	0.91
